# Phosphorylation of estrogen receptor α at serine 118 is correlated with breast cancer resistance to tamoxifen

**DOI:** 10.3892/ol.2013.1324

**Published:** 2013-04-29

**Authors:** MING CHEN, YU-KUN CUI, WEN-HE HUANG, KWAN MAN, GUO-JUN ZHANG

**Affiliations:** 1 Breast Center of The Affiliated Cancer Hospital of Shantou University Medical College, Shantou, Guangdong 515031;; 2 Department of Obstetrics and Gynecology, Peking Union Medical College Hospital, Chinese Academy of Medical Sciences and Peking Union Medical College, Beijing 100730;; 3 The Central Laboratory of The Affiliated Cancer Hospital of Shantou University Medical College, Shantou, Guangdong 515031;; 4 Department of Surgery, Li Ka-Shing Faculty of Medicine, Hong Kong University, Hong Kong, SAR;; 5 Cancer Research Center, Shantou University Medical College, Shantou, Guangdong 515041, P.R. China

**Keywords:** estrogen receptor α, phosphorylation, tamoxifen, breast cancer, endocrine resistance

## Abstract

The aim of the present study was to explore the correlation between estrogen receptor α (ERα) phosphorylation at serines 118 and 167 and the responsiveness of patients with primary breast cancer to tamoxifen. Tumors from 104 patients with primary breast cancer who received adjuvant tamoxifen therapy at The Affiliated Cancer Hospital of Shantou University Medical College between January 2001 to December 2007 were subjected to immunohistochemical analysis with specific antibodies against ERα phosphorylated at either serine 118 (pERα-S118) and/or serine 167 (pERα-S167). ERα phosphorylation at the two sites was correlated with either the disease-free survival or the overall survival rate of these patients using the Kaplan-Meier survival analysis. pERα-S118 and pERα-S167 were found to be expressed in the cell nucleus of 25.0% (26/104) and 26.9% (28/104) of breast cancers, respectively. The expression of pERα-S118 was positively correlated with the human epidermal growth factor receptor-2 (HER-2) status (χ^2^=6.85, P=0.01). The Kaplan-Meier analysis revealed a poorer disease-free (P=0.022) and overall survival (P=0.013) in breast cancer patients expressing pERα-S118, but not in those expressing pERα-S167. In conclusion, pERα-S118 was correlated with the HER-2 status and predicted breast cancer resistance to tamoxifen.

## Introduction

Breast cancer is the most frequently detected female neoplasm worldwide. The estrogen receptor (ER) is expressed by 60–70% of breast tumors (1); therefore, targeting the ER has emerged as a major management technique for ER-positive breast cancers. Tamoxifen is the most commonly used antiestrogen, but resistance remains the major obstacle for its clinical application. While up to one-third of patients are resistant to tamoxifen at the beginning of treatment, the majority of patients who initially respond to tamoxifen will later also become resistant (2). Although the mechanisms underlying tamoxifen resistance are largely unknown, increasing evidence has indicated that ERα cross-communicates with growth factor signaling, including epidermal growth factor receptor (EGFR) and mammalian target of rapamycin (mTOR) signaling, and the resultant ERα phosphorylation, are important in tamoxifen resistance (3).

As a ligand-activating transcription factor, ERα has been found to be phosphorylated at numerous sites, including serines 118, 167, and 305 (4). These three sites have been demonstrated to be phosphorylated by extracellular signal-regulated kinase/mitogen-activated protein kinase (ERK/MAPK), protein kinase B (Akt) and protein kinase A (PKA) and/or p21-activated protein kinase (Pak1), respectively, and appear to be the most relevant sites with regard to breast cancer resistance to tamoxifen (4). Certain studies have demonstrated that the reduced ERα phosphorylation at serine 118 (pERα-S118) and the increased ERα phosphorylation at serine 167 (pERα-S167) were correlated significantly with the improved disease-free survival (DFS) and overall survival (OS) of breast cancer patients, while alternative studies have suggested a correlation between either pERα-S118 or pERα-S167 and tamoxifen resistance (5). The effects of pERα-S118 and pERα-S167 have not yet been elucidated, and therefore further studies are required.

In the present study, the status of pERα-S118 and pERα-S167 proteins were immunohistochemically detected in breast tumors from patients who received adjuvant tamoxifen treatment, and the clinicopathological features, the DFS and the OS of these patients were correlated with the status of either pERα-S118 or pERα-S167. The results showed that ERα phosphorylation was correlated with human epidermal growth factor receptor-2 (HER 2) status at serine (Ser) 118, but not Ser 167, and also predicted the resistance of breast cancer to tamoxifen.

## Materials and methods

### Patients and breast cancer tissues

Breast tumor specimens from 104 female patients with invasive breast carcinoma, who had registered at The Affiliated Cancer Hospital of Shantou University Medical College between January 2001 and December 2007, were included in the present study. The study protocol was approved by the institutional review board of Shantou University Medical College (Guangdong, China). Written informed consent was obtained from all patients. All patients had undergone surgical treatments for primary breast cancer (either mastectomy or lumpectomy), and all primary tumors were ERα-positive, as defined by immunohistochemical staining. The samples were selected from a continuous series of invasive carcinoma tissues. Following surgery, 78 patients received systemic adjuvant chemotherapy and 47 received radiotherapy. All patients received 10 mg tamoxifen twice a day as an endocrine therapy, either until the disease had progressed or for five years, following the previously mentioned treatments. The follow-up time ranged from 50–121 months.

### Immunohistochemical analysis

A 4-*μ*m section of each submitted paraffin block was first stained with hematoxylin and eosin to verify that an adequate number of invasive carcinoma cells were present. Serial sections (4 *μ*m) were prepared from selected blocks and float-mounted onto adhesive-coated glass slides. In order to stain for pER*α*-Ser118/167, the slides were oven-boiled in a citrate buffer (pH 6.0) for 20 min, cooled at room temperature for 20 min and then placed into methanol containing 3% H_2_O_2_ for 5 min to inactivate the endogenous peroxidase. This was followed by 10 min of incubation with a serum-free protein block solution (DakoCytomation, Glostrup, Denmark). The slides were subsequently washed three times and incubated with 1:100 diluted anti-rabbit polyclonal pER*α-*S118/167 antibodies (Cell Signaling Technology, Inc., Boston, MA, USA). Following this, the slides were placed in a moisturized chamber at 4°C for 20 h. The slides were then washed, incubated with the anti-rabbit DakoCytomation Envision+ system for 30 min at 4°C, visualized with 3,3′-diaminobenzidine hydrochloride in phosphate buffer containing 0.03% H_2_O_2_ and counterstained with hematoxylin. All washing steps were performed in phosphate-buffered saline solution with 0.5% bovine serum albumin. The staining intensity was evaluated on three separate biopsies for each tumor and was scored by two trained histopathologists, using the modified McCarty’s H-scoring system (6). This utilized the percentage of positive cells and the intensity of staining to provide a total score, varying from 0–300. The staining was designated as negative (−; H-score, <50), weakly positive (+; H-score, 51–100), moderately positive (++; H-score, 101–200) or strongly positive (+++; H-score, 201–300) As the proportion of positive staining was moderate, the cutoff point for positive staining was set to ≥1% in the statistical analysis. pER*α*-S118/167 was occasionally visible in the cytoplasm, but only nuclear staining was graded.

### Statistical analysis

The χ^2^ test was used to compare the expression of pER*α*-S118/167 with the clinicopathological characteristics. The student’s t-test was used to compare the survival time between the uni- and coexpression of pER*α*-S118/167. An estimation of patient survival was performed using the Cox regression method, and a Kaplan-Meier curve was used to assess the survival differences between the pER*α*-S118-postive and -negative patients, as well as between the pER*α*-S167-positive and -negative samples. P<0.05 was considered to indicate a statistically significant difference.

## Results

### Correlation between pERα-S118 or pERα-S167 expression and clinicopathological factors in primary breast tumors

**Figure 1.**
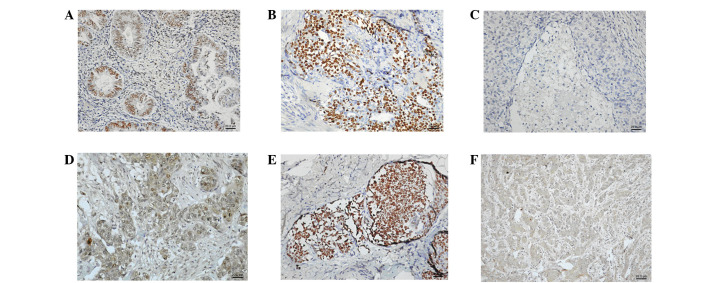
Immunohistochemical results of estrogen receptor α phosphorylation at serine 118 (pERα-S118) and 167 (pERα-S167) in patients with primary breast cancer. (A) pERα-S118 (+); (B) pERα-S118 (+++); (C) pERα-S118 (−); (D) pERα-S167 (+); (E) pERα-S167 (+++); and (F) pERα-S167 (−). Magnification, x200. −, negative; +, weakly positive; +++, strongly positive.

pERα-S118 and/or pERα-S167 were immunohistochemically detected in a total of 104 primary invasive breast carcinomas, as demonstrated in Fig. 1. A total of 25.0% (26/104) of carcinomas were positive for pERα-S118 (Figs. 1A–C), whereas 26.9% (28/104) were positive for pERα-S167 (Figs. 1D–F). All graded phosphorylation staining occurred in the nucleus.

**Figure 2.**
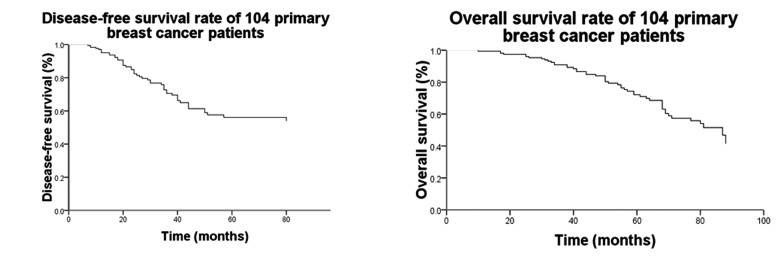
Kaplan-Meier graphs for the disease-free and overall survival rates of 104 primary breast cancer patients undergoing tamoxifen therapy.

The DFS and OS values of 104 primary breast cancer patients are shown in Fig 2, whilst the correlation between the clinicopathological factors and the survival of the patients is shown in Table I. The factors affecting the DFS and OS were the tumor size and axillary lymph node staging. DFS was also shown to be affected by the HER-2 status (P=0.041). No correlation was observed between pERα-S118/167 and survival time.

The immunohistochemistry (IHC) scores for pERα-S118 and pERα-S167 were correlated with various clinicopathological factors. The clinicopathological factor distribution between the positive and negative expression of pERα-S118 and pERα-S167 is demonstrated in Table II. A significant positive correlation was found between pERα-S118 expression and HER-2 status, as determined by IHC (χ^2^=6.85, P=0.01). Among the 26 pERα-S118-positive patients, 20 were HER-2 positive, while out of the 76 pERα-S118-negative patients, 37 were HER-2 positive. In contrast, no correlation was identified between pERα-S167 and the HER-2 status (χ^2^= 0.23, P= 0.63). There was also no significant difference observed between pERα-S118/pERα-S167 and the tumor size (χ^2^=2.02, P=0.16 and χ^2^=1.88, P=0.17, respectively) or lymph node status (χ^2^= 0.02, P= 0.90 and P= 0.09, χ^2^=2.80, respectively). In addition, there was no correlation between pERα-S118/167 and the progesterone receptor status (χ^2^= 0.95, P=0.33 and χ^2^=1.56, P=0.21, respectively), the age at diagnosis (χ^2^=0.83, P=0.36 and χ^2^= 0.85, P= 0.36, respectively) and the menopausal status (χ^2^=0.12, P=0.73 and χ^2^=3.00, P=0.08, respectively).

### pERα-S118 may predict the resistance of breast cancer to tamoxifen

**Figure 3.**
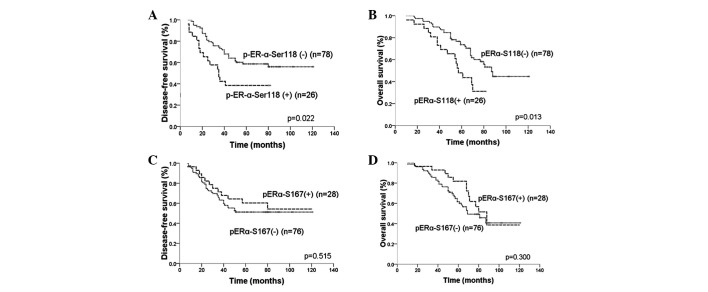
Kaplan-Meier graphs for the effect of the phosphorylation of estrogen receptor α at serine 118 (pERα-S118) and 167 (pERα-S167) on (A,C) disease-free and (B,D) overall survival. (A) χ^2^=5.218 and P=0.022; (B) χ^2^=6.216 and P=0.013; (C) χ^2^=0.424 and P=0.515; and (D) χ^2^=1.075 and P=0.300.

The primary aim of the present study was to determine whether pERα-S118 or pERα-S167 were related to the clinical outcome in patients treated with tamoxifen. pERα-S118/S167 was determined to be a binary factor (positive where detectable nuclear staining was present and negative where detectable nuclear staining was absent) and a Kaplan-Meier survival analysis was performed since fewer than one-third of the samples exhibited positive staining for either pERα-S118 or pERα-S167. Kaplan-Meier plots of DFS and OS are shown in Fig. 3. Those patients whose primary tumors expressed pERα-S118 had a shorter DFS and OS than those whose tumors were pERα-S118-negative (P=0.022 and P= 0.013, respectively; Figs. 3A and B). Although there was an apparent correlation between pERα-Ser167 and OS (Figs. 3C and D), the results were not significant (P=0.515 and P=0.300, respectively; Fig. 3). This was most likely due to the small number of events (fatalities) occurring in the present study.

Among the 104 patients, there were seven samples expressing positive staining for pERα-S118 and pERα-S167. A comparison between these seven samples and those samples exhibiting positive staining for either pERα-S118 or pERα-S167 indicated that the uni-/coexpression of pERα-S118/pERα-S167 had no effect on DFS and OS (Table III).

## Discussion

Although third generation aromatase inhibitors exhibit certain advantages over tamoxifen in postmenopausal breast cancer patients, tamoxifen remains the first-line treatment for numerous ER-positive breast cancer patients. Approximately one-third of ER-positive tumors will gradually develop resistance to endocrine therapy and, in particular, to tamoxifen treatment (2). The determination of the prognostic factor(s) for breast cancer patients treated with tamoxifen may aid in the optimization of the therapeutic strategy and also in overcoming the resistance to endocrine therapy.

It has been demonstrated that ER-α may be phosphorylated on multiple amino acid residues (4). In general, phosphorylation in the activation function-1 (AF1) domain of ERα appears to recruit coactivators, resulting in enhanced ERα-mediated transcription, and also affect the cellular response to tamoxifen (7). In the present study, the nuclear expression of pERα-S118 and pERα-S167 was detected by IHC and the correlation with tamoxifen responsiveness was also analyzed.

It was identified that pERα-S118 and pERα-S167 were expressed in a proportion of tamoxifen-resistant breast tumors, and that their expression did not exhibit any correlation with the age, menopausal status, TNM (tumor size, axillary lymph node staging) stage or ER and PR status of the patient. However, pERα-S118, but not pERα-S167, was significantly correlated with the expression level of HER-2 and with a shorter survival time in breast cancer patients. The HER-2 levels are amplified in ∼20% of breast cancer patients and HER-2 overexpression is associated with a poorer prognosis (8). The HER-2 gene is located on the 17q chromosome and encodes a transmembrane tyrosine growth receptor that produces a protein receptor on the cell membrane with a molecular weight of ∼185 kDa (9). It has been confirmed that HER-2 may be correlated with the advanced progression and poorer prognosis of breast cancer. Cittelly *et al* demonstrated that breast cancer overexpressing HER-2 exhibited resistance to tamoxifen through the upregulation of B-cell CLL/lymphoma 2 (BCL-2) and the suppression of miR-15a/16 induced by tamoxifen (10). Yamashita *et al* indicated that pERα-S118 was positively and significantly correlated with HER-2 and that it was amplified in the AIB1 gene (11). Certain studies have revealed that ERα may be phosphorylated at Ser 118 by ERK-MAPK (12,13). ERK-MAPK is a downstream kinase of HER-2 (14), and thus the HER2-MAPK-AIB1-pERα-S118 pathway may form a kinase cascade that leads to a poorer prognosis when the pathway is activated. Further studies into this mechanism are required.

Most notably, the present analysis has demonstrated that pERα-S118 is correlated with poorer survival. pERα-S118 was associated with a shorter DFS and OS (P=0.022 and P= 0.013, respectively), however, there was no statistically significant correlation between pERα-S167 and the DFS or OS of the breast cancer patients (P=0.515 and P=0.300, respectively). Previous studies *in vivo* and *in vitro* remain divided over whether pERα-S167 or pERα-S118 have an impact on tamoxifen resistance (11,15–18). Sarwar *et al* demonstrated that Ser 118 phosphorylation was elevated in tumor biopsies taken from patients who had relapsed following tamoxifen treatment (19). Yamashita *et al* revealed that a higher expression of pERα-S118 was a predictor of poorer survival, which is consistent with the present study (11). By contrast, Murphy *et al* analyzed pERα-S118 by IHC in 117 breast cancer tissues and demonstrated that it was a marker of improved prognosis in patients treated with tamoxifen (20). However, in the study by Murphy *et al*, the determination of ERα-positive tumors was analyzed by ligand binding assay, not by IHC, and their patient inclusion criteria comprised axillary lymph node-negative, and not only ERα-positive, tumors. Other studies have also demonstrated that pERα-S167 has an effect on the survival of breast cancer patients. Yamashita *et al* indicated that a higher expression of pERα-S167 was correlated with improved survival in ER-positive breast cancers (11). However, Guo *et al* demonstrated that pERα-Ser167 was phosphorylated by inhibitor of kappa B kinase-ε (IKKε) *in vitro* and *in vivo*, leading to the upregulation of cyclin D1 and resulting in tamoxifen resistance (16). In the present study, however, we failed to demonstrate any correlation between pERα-S167 and tamoxifen resistance. There may be several reasons for these discrepancies; for example, there were varying detection methods and cutoff points for diagnosis, and so uniform standards of detection should be discussed and investigated in the future. In addition, there were differing inclusion criteria for the samples. Although certain studies took biopsies (17,21), a number of studies used samples following surgery (11,17,20). Moreover, there were differences in the treatment that the patients received following surgery. The patients in the present study received chemotherapy and/or radiation according to National Comprehensive Cancer Network (NCCN) guidelines. However, in the study by Yamashita *et al*, patients with positive axillary lymph nodes did not receive radiation (11). It is therefore difficult to estimate the true effect of tamoxifen therapy in post-operative adjuvant therapy. Furthermore, the effect of ethnicity remains unclear.

Combinatorial regulation has been discussed in numerous biochemical phenomena, including the cophosphorylation of various sites in ERα (22). In the present study, the coexpression of pERα-S118 and pERα-S167 was detected in seven out of the 104 samples (6.73%). The DFS and OS of these patients were similar to that of patients solely expressing pERα-S118 (P= 0.79 and 0.87, respectively). The OS was significantly different from that of patients solely expressing pERα-S167 (P= 0.05), suggesting that pERα-S167 may not be a significant predictor for breast cancer responsiveness to tamoxifen. However, the DFS was not significantly different from that of patients solely expressing pERα-S167 (P=0.26). As there were only seven cases of coexpression in the present study, a larger sample size is required in future studies.

In conclusion, the present study found that adjuvant tamoxifen-treated breast cancer patients who have primary tumors expressing pERα-S118 have a shorter DFS and OS. The data suggest that pERα-S118 may be an indicator for the resistance of breast cancer to tamoxifen.

## Figures and Tables

**Table I.** Correlation between clinicopathological factors, DFS and OS in 104 primary breast tumors of luminal type.

Clinicopathological factors	No. of cases	DFS		OS	
		Wald statistic	P-value	Wald statistic	P-value
Age (years)		4.786	0.680	5.865	0.566
≤50	48				
>50	56				
Menopausal status		2.735	0.541	3.100	0.432
Premenopausal	59				
Postmenopausal	45				
T		4.313	0.040	2.495	0.023
T1	19				
T2	57				
T3	24				
T4	4				
N		17.467	0.002	23.220	0.000
N0	48				
N1	27				
N2	17				
N3	12				
ER		3.133	0.372	4.319	0.229
−	3				
+	10				
++	25				
+++	66				
PR		1.427	0.699	2.051	0.562
−	23				
+	23				
++	23				
+++	35				
HER-2		4.174	0.041	2.598	0.107
Negative	47				
Postive	57				
p-ERα-S118		1.686	0.194	2.882	0.090
Negative	78				
Postive	26				
p-ERα-S167		1.738	0.187	0.989	0.320
Negative	76				
Postive	28				

DFS, disease-free survival; OS, overall survival; T, tumor size; N, axillary lymph node staging; ER, estrogen receptor; PR, progesterone receptor; HER-2, human epidermal growth factor receptor 2; p-ERα-S118, estrogen receptor α phosphorylation at serine 118; p-ERα-S167, estrogen receptor α phosphorylation at serine 167;

−, negative;

+, weakly positive;

++, moderately positive;

+++, strongly positive.

**Table II.** Correlation between expression of pERα-Ser118 and pERα-Ser167 and the clinicopathological factors in primary breast tumors.

Clinicopathological factors	pERα-Ser118				pERα-Ser167			
	+, n (%)	−, n (%)	χ^2^	P-value	+, n (%)	−, n (%)	χ^2^	P-value
T								
T1 and T2	22 (28.57)	55 (71.43)	2.02	0.16	23 (30.26)	53 (69.74)	1.88	0.17
T3 and T4	4 (14.81)	23 (85.19)			5 (17.86)	23 (82.14)		
N								
N0 and N1	19 (25.33)	56 (74.67)	0.02	0.90	17 (22.67)	58 (77.33)	2.80	0.09
N2 and N3	7 (24.14)	22 (75.86)			11 (37.93)	18 (62.07)		
PR								
Positive	20 (24.69)	61 (75.31)	0.95	0.33	22 (27.16)	59 (72.84)	1.56	0.21
Negative	6 (26.09)	17 (73.91)			6 (26.09)	17 (73.91)		
HER-2								
Positive	20 (35.09)	37 (64.91)	6.85	0.01	15 (26.32)	42 (73.68)	0.23	0.63
Negative	6 (12.77)	41 (87.23)			13 (27.66)	34 (72.34)		
Age (years)								
<50	10 (20.83)	38 (79.17)	0.83	0.36	15 (31.25)	33 (68.75)	0.85	0.36
≥50	16 (28.57)	40 (71.43)			13 (23.21)	43 (76.79)		
Menopausal status								
Premenopausal	14 (23.73)	45 (76.27)	0.12	0.73	12 (20.34)	47 (79.66)	3.00	0.08
Postmenopausal	12 (26.67)	33 (73.33)			16 (35.56)	29 (64.44)		

pERα-S118, estrogen receptor α phosphorylation at serine 118; pERα-S167, estrogen receptor α phosphorylation at serine 167; T, tumor size; N, axillary lymph node staging; PR, progesterone receptor; Age, age at diagnosis; HER-2, human epidermal growth factor receptor 2.

**Table III.** Correlation between pERα-S118/167 and the DFS and OS times.

	Number	DFS (months)	P-value	OS (months)	P-value
pERα-S118 (+)	19	39.68±6.11	0.79	53.42±4.75	0.87
pERα-S167 (+)	21	64.90±7.46	0.26	76.62±5.61	0.05
Both (+)	7	41.71±10.00		53.86±7.68	
Both (−)	57	55.32±3.67		63.65±2.95	

DFS and OS are presented as mean ± SD. P-value; comparison with the Both (+) group. DFS, disease-free survival; OS, overall survival; pERα-S118, estrogen receptor α phosphorylation at serine 118; pERα-S167, estrogen receptor α phosphorylation at serine 167; (+), positive expression; (−) negative expression.
